# Classifying Three-Wall Intrabony Defects from Intraoral Radiographs Using Deep Learning–Based Convolutional Neural Network Models

**DOI:** 10.1055/s-0044-1791784

**Published:** 2024-11-21

**Authors:** Kanteera Piroonsan, Kununya Pimolbutr, Kallapat Tansriratanawong

**Affiliations:** 1 Department of Oral Medicine and Periodontology, Faculty of Dentistry, Mahidol University, Bangkok, Thailand

**Keywords:** deep learning, machine learning, neural networks, artificial intelligence, periodontal bone loss, intrabony defect

## Abstract

**Objective** Intraoral radiographs are used in periodontal therapy to understand interdental bony health and defects. However, identifying three-wall bony defects is challenging due to their variations. Therefore, this study aimed to classify three-wall intrabony defects using deep learning–based convolutional neural network (CNN) models to distinguish between three-wall and non-three-wall bony defects via intraoral radiographs.

**Materials and Methods** A total of 1,369 radiographs were obtained from 556 patients who had undergone periodontal surgery. These radiographs, each featuring at least one area of intrabony defect, were categorized into 15 datasets based on the presence of three-wall or non-three-wall intrabony defects. We then trained six CNN models—InceptionV3, InceptionResNetV2, ResNet50V2, MobileNetV3Large, EfficientNetV2B1, and VGG19—using these datasets. Model performance was assessed based on the area under curve (AUC), with an AUC value ≥ 0.7 considered acceptable. Various metrics were thoroughly examined, including accuracy, precision, recall, specificity, negative predictive value (NPV), and F1 score.

**Results** In datasets excluding circumferential defects from bitewing radiographs, InceptionResNetV2, ResNet50V2, MobileNetV3Large, and VGG19 achieved AUC values of 0.70, 0.73, 0.77, and 0.75, respectively. Among these models, the VGG19 model exhibited the best performance, with an accuracy of 0.75, precision of 0.78, recall of 0.82, specificity of 0.67, NPV of 0.88, and an F1 score of 0.75.

**Conclusion** The CNN models used in the study showed an AUC value of 0.7 to 0.77 for classifying three-wall intrabony defects. These values demonstrate the potential clinical application of this approach for periodontal examination, diagnosis, and treatment planning for periodontal surgery.

## Introduction

Periodontal disease progression occurs by microbial dysbiosis of subgingival pathogens, and host defense mechanisms leading to clinical attachment loss and alveolar bone resorption. 1 This process may eventually create variable defects in the alveolar bone, such as suprabony and intrabony defects. 2 3 Patterns of intrabony defects are classified based on the remaining bony walls, which influence decision-making, diagnosis, prognosis, and treatment planning in periodontal therapy. 4 5 6 Evaluating intrabony sites is critical to successful periodontal surgery, especially for regenerative osseous procedures, which have superior regenerative potential with additional remaining bony walls. 7 To achieve accurate prediction results, it is essential to correctly identify and assess intrabony defects, specifically three-wall bony defects.

Bone sounding and radiographic examinations are two common methods used to assess bony defects. 8 9 Bone sounding is a procedure that determines the depth, angle, and shape of an intrabony defect, but it can be painful and uncomfortable for patients. 10 On the other hand, detecting three-wall intrabony defects from two-dimensional intraoral radiographs can be challenging due to variable factors. 11 12 13 Although cone-beam computed tomography (CBCT) is highly accurate in diagnosing intrabony defects, it is not commonly used because of its cost and potential radiation exposure. 14 Some computer-assisted methods that use linear or subtraction measurements have also been tried, but they are often less accurate and inconsistent compared to surgical evaluation. 15 16

Digital dentistry, including artificial intelligence (AI), is transforming oral health practices and dental education. Deep learning (DL), a subset of AI that mimics the human brain to solve problems, has been widely used in dentistry. The convolutional neural network (CNN) technique is particularly popular, as it allows for precise identification and categorization of images. 17 18 CNNs are capable of automatically extracting important features from images, which leads to accurate detection and reduces processing costs and time. Therefore, CNNs are widely used for image classification. Recently, CNNs have been used for diagnosis and prognosis in periodontal therapy to detect alveolar bone loss with high accuracy. 19 Lee et al used CNNs to evaluate periodontally compromised teeth for periodontal diagnosis and prognosis, and the accuracy of the model was between 0.734 and 0.828 in prediction tasks. 20 Panoramic films are typically used with CNNs to determine the alveolar bone loss in periodontal diagnosis, but the model's performance varies and ranges from approximately 0.77 to 0.98 for different tooth positions and model classifications. 21 22 23 24 Bone loss estimation from periapical radiographs has reached an accuracy of approximately 0.81 to 0.97. 25 26 27 By minimizing the need for invasive procedures like bone sounding, AI-based models can enhance precision in diagnosis, thus reducing the consumption of physical materials and promoting efficient treatments. However, studies identifying intrabony defects, particularly three-wall bony defects via intraoral radiographs, lack evidence. While previous studies have shown that CNNs can detect alveolar bone loss with similar accuracy to clinician interpretation, evaluating intrabony defects requires high skill and experience from clinicians.

Therefore, CNNs can provide dentists with technology-based diagnostic tools that are highly accurate and require less time. Moreover, they can be used in the periodontal field for precise classification, detection, and measurement of periodontal bone levels and defects. Thus, this study aimed to classify three-wall intrabony defects using DL-based CNN models to distinguish between three-wall and non-three-wall bony defects via intraoral radiographs.

## Materials and Methods

### Ethical Approval Statement

The Institutional Review Board, Faculty of Dentistry/Faculty of Pharmacy, Mahidol University (MU-DT/PY-IRB 2023/019.2102) approved the study.

### Study Population and Data Collection

The individuals of interest were identified from the periodontal surgery list at the Periodontics Clinic, Faculty of Dentistry, Mahidol University, and Maha Chakri Sirindhorn Dental Hospital from January 2013 to December 2022.

**Fig. 1:**
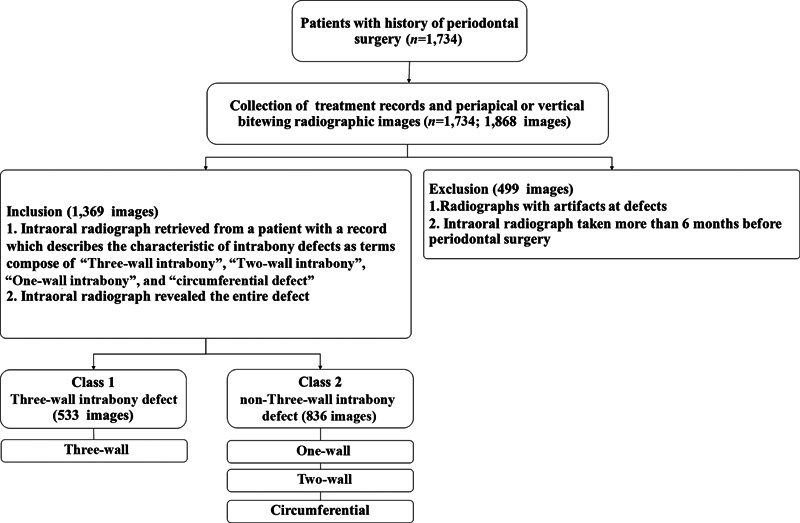
Flowchart of the study of population. A total of 1,734 subjects with intraoral radiographs were evaluated for the bony defects of previously periodontal surgery and classified into two classes: class 1 three-wall intrabony defect and class 2 non-three-wall intrabony defect.

A total of 1,734 patients with a history of periodontal surgeries (e.g., flap surgery, osseous surgery, and regeneration with intrabony morphological measures) were identified from the data sources. Patients who underwent intraoral (periapical and/or vertical bitewing) radiographic examination revealing areas of interest were recruited. These radiographs were reviewed by inclusion and exclusion criteria ( Fig. 1 ).

Demographic data such as age, sex, type of tooth, number of bony walls, and type of periodontal surgery were collected retrospectively from patients' medical records. The eligible radiographs displaying interest areas were retrieved from the picture archiving and communication system (PACS), examined, and exported in JPEG format.

### Data Preparation

**Fig. 2:**
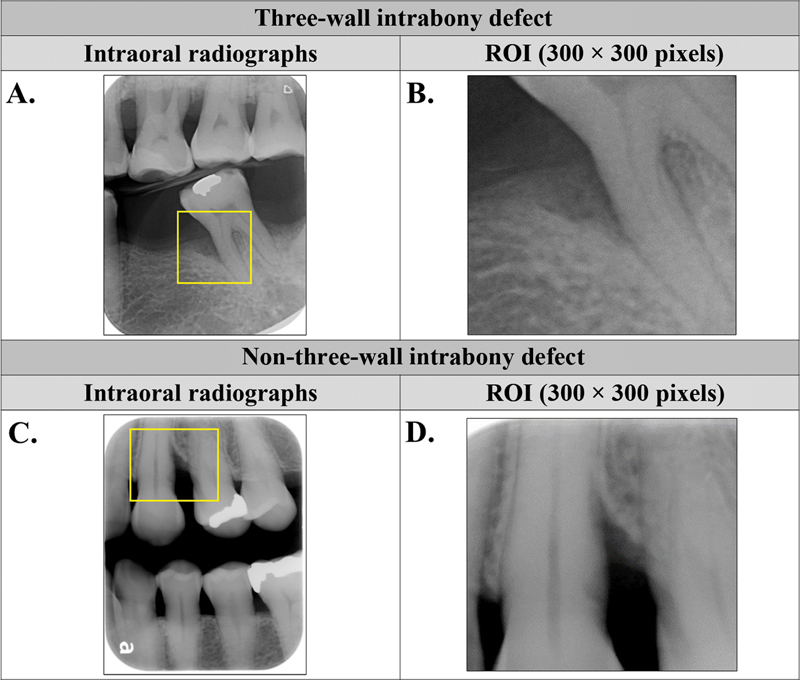
Images demonstrated representative vertical bitewing radiographs, which categorized into three-wall (2A, B) and non-three-wall bony defects (2C, D). Original images (2A, C) were cropped into the ROI (yellow rectangular box) at 300 × 300 pixels (2B, D). ROI, region of interest.

**Fig. 3:**
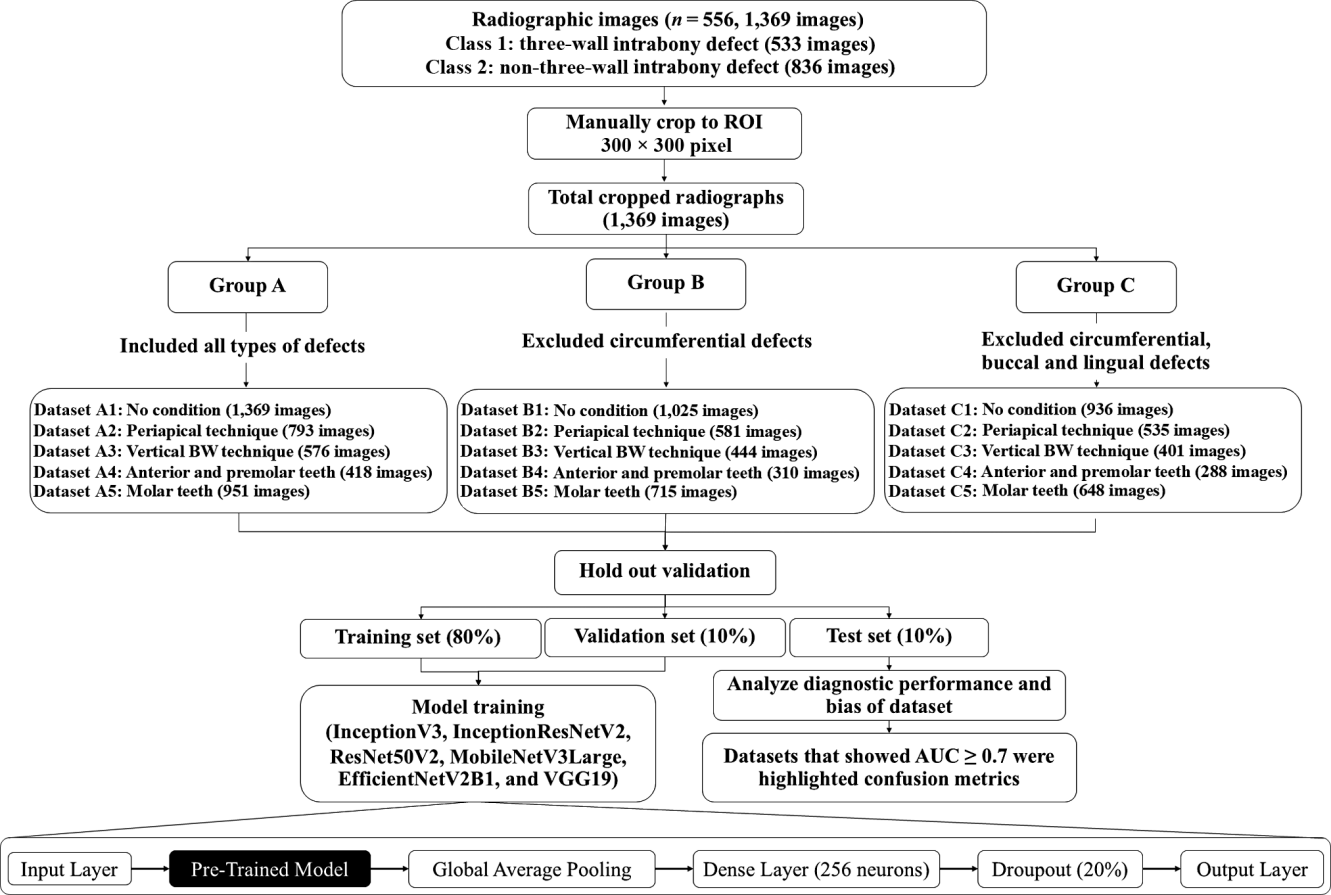
Flowchart of the dataset preparation, data preprocessing, and model training. A total of 1,369 cropped images were classified into three group (groups A–C) based on specific sites of defect; group A included all defects, group B excluded circumferential defects, and group C excluded both circumferential defects and buccal or lingual defects. In addition, all groups were further divided into five categories according to radiographic technique and tooth type: (1) no condition, (2) periapical technique, (3) vertical bitewing (BW) technique, (4) anterior and premolar teeth, and (5) molar teeth. A total of 15 datasets (A1–5, B1–5, and C1–5) were established for enhancing the model's potential. The holdout validation method was used for training and validation of six selected CNN models: InceptionV3, InceptionResNetV2, ResNet50V2, MobileNetV3Large, EfficientNetV2B1, and VGG19. Model performances were evaluated by the area under the receiver operating characteristic curve (AUC), which was considered acceptable at an AUC value ≥ 0.7. AUC, area under curve; BW, bitewing; CNN, convolutional neural network; ROI, region of interest.

The eligible radiographs were classified into three-wall (class 1; Fig. 2A, B ) and non-three-wall (class 2; Fig. 2C, D ) bony defect, and manually cropped in the region of interest (ROI), which consisted of the bony defect adjacent to the tooth and was centrally located within the radiographs. The cropping process was performed using PhotoScape X 4.2.1 (MOOII Tech, Korea), and the cropped images were resized to dimensions 300 × 300 pixels ( Fig. 2B, D ). A total of 1,369 cropped radiographs were classified into three groups: group A included all defects, group B excluded circumferential defects, and group C excluded both circumferential defects and buccal or lingual defects. In each group, data were created based on specific sites and configurations of bony walls for enhancing the model's potential, as illustrated in Fig. 3 .

Groups A to C were further divided into subgroups or datasets based on radiographic technique and tooth type to five specific conditions: (1) no condition, (2) periapical technique, (3) vertical bitewing technique, (4) anterior and premolar teeth, and (5) molar teeth. This division resulted in a total of 15 datasets (datasets A1–5, B1–5, and C1–5; Fig. 3 ).

### Data Preprocessing

In this phase, all subsequent steps until model evaluation were performed using Python (version 3.9.5), with TensorFlow (version 2.10.1, United States) as the main DL library, utilizing the computational power of an RTX3050 GPU (NVIDIA GeForce RTX3050, California, United States). The images obtained from the previous data preparation stage were divided into three separate datasets, following a hold-out validation method, resulting in a training dataset, validation dataset, and test dataset at a ratio of 8:1:1 ( Fig. 3 ).

### CNN Model Training

Pretrained models from ImageNet (2020 Stanford Vision Lab, Stanford University, Princeton University, United States) were implemented. The selected models, namely, InceptionV3, InceptionResNetV2, ResNet50V2, MobileNetV3Large, EfficientNetV2B1, and VGG19, underwent training with image datasets. To adapt these pretrained models to the specific task of distinguishing between three-wall and non-three-wall intrabony defects, the top layers of the models were replaced with a newly constructed configuration of layers. The modified top layers comprised a global average pooling layer, dense layers featuring 256 units, a dropout layer with a 20% dropout rate, and an output layer with two nodes representing the two defect categories (classes 1 and 2; Fig. 3 ). The training procedure involved two main datasets: the training dataset for model training and the validation dataset for model validation. During training, the categorical cross-entropy loss function and the Adam optimizer were utilized. 28 The training process was conducted with a batch size of 32 (except for VGG19, for which the batch size was 16), 150 epochs, and a learning rate 0.0001.

### Statistical Analysis

The area under the receiver operating characteristic (ROC) curve (area under curve [AUC]) and confusion metric were utilized to assess the model's classification performance. The AUC is a pivotal metric used to assess the overall predictive capacity of models for distinguishing three-wall and non-three-wall intrabony defects. As noted by Hosmer and Lemeshow, an AUC value of 0.5 indicates minimal discrimination ability, while AUC values of 0.7 to 0.8, 0.8 to 0.9, and ≥ 0.9 are considered acceptable, excellent, and outstanding, respectively. 29 If the AUC from training based on the total radiograph dataset did not exceed 0.7 in any of the models, the datasets were created and narrowed down under specific conditions. Moreover, the positive predictive value (PPV/precision), sensitivity (recall), specificity, negative predictive value (NPV), and F1 score (the harmonic mean of precision and recall) were calculated from the confusion matrices as the formulas below:



where TP is true positive, TN is true negative, FP is false positive, and FN is false negative.

The demographic, clinical, and radiographic data were presented as frequencies and percentages for categorical data. For normally distributed continuous variables, the mean and standard deviation were used as summary statistics (Microsoft Excel in Microsoft 365 version 2308).

## Results

### Demographic Data

After screening, a total of 1,369 radiographs were included in the study; 578 (42.2%) were bitewing radiographs, and 791 (57.8%) were periapical radiographs. Radiographs were obtained from 556 patients, 303 (54%) females and 253 (46%) males. The mean age of all participants was 59.48 ± 10.68 years. The distributions of tooth type and the location of the defect are reported in Table 1 . Defect type was 39% three-wall group and 61% non-three-wall group.

**Table 1:** Characteristic of patient, tooth type, and radiologic data

Patients	*N* (%)
Number of females	303 (54%)
Number of males	253 (46%)
Total patients	556 (100%)
Average age (years ± SD)	59.48 ± 10.68
Radiographic images	Images (%)
Total images	1369 (100%)
Technique	
Periapical	791 (58%)
Bite wing	578 (43%)
Tooth type	
Anterior	117 (9%)
Premolar	301 (22%)
Molar	951 (70%)
Area	
Maxilla	557 (41%)
Mandibular	792 (58%)
Surface	
Mesial	671 (49%)
Distal	467 (34%)
Buccal	104 (8%)
Lingual	127 (9%)
Defect type	
Class 1 (three-wall group)	533 (39%)
Class 2 (Non-three-wall group)	836 (61%)

### Dataset Selection

The evaluation of the model's performance based on the total radiographs (dataset A1) revealed a range of AUC values from 0.44 to 0.64. Five conditions (datasets B1, B3, C1, C3, and C4) yielded acceptable results, with AUC values ranging between 0.7 and 0.79, as described in Table 2 .

**Table 2:** AUC from each model training using different datasets

Dataset	Radiographic images	Description	InceptionV3	InceptionResNetV2	ResNet50V2	MobileNetV3Large	EfficientNetV2B1	VGG19
A1	1,369	Total radiographs	0.64	0.56	0.60	0.59	0.44	0.56
B1	1,025	Excludes circumferential defects	0.69	0.64	0.63	0.62	0.54	0.72
B3	444	Excludes circumferential defects, includes bitewing radiographs	0.66	0.70	0.73	0.77	0.59	0.75
C1	936	Excludes circumferential defects, buccal/lingual surface defects	0.73	0.71	0.64	0.68	0.59	0.65
C3	401	Excludes circumferential defects, buccal/lingual surface defects, includes bitewing radiographs	0.71	0.47	0.74	0.61	0.64	0.52
C4	288	Excludes circumferential defects, buccal/lingual surface defects, includes anterior and premolar teeth	0.79	0.79	0.73	0.63	0.55	0.57

Abbreviation: AUC, area under curve.

When dataset B3 further enhanced model performance, AUC values ranging from 0.7 to 0.77 were obtained with four models (e.g., MobileNetV3Large, VGG19, RestNet50V2, and InceptionResNetV2). InceptionV3 and RestNet50V2 displayed AUC values of 0.71 and 0.73, respectively, when training with dataset C1. On the other hand, dataset C3 produced varying improvements, with AUC values ranging from 0.47 to 0.74. Notably, ResNet50V2 achieved the highest AUC value (0.74). Furthermore, dataset C4 had the highest AUC value (0.79) when combined with InceptionV3 and InceptionResNetV2 ( Table 2 ).

These datasets, which generally met the acceptable AUC range criteria, confirmed the model's effectiveness in distinguishing and classifying the specified intrabony defects. Based on the model evaluation results, dataset B3 was the optimal choice for data selection. The model performance improved significantly compared to the baseline dataset, with AUC values ranging from 0.59 to 0.77. Notably, MobileNetV3Large achieved the highest AUC value of 0.77 for dataset B3, and VGG19 displayed a competitive AUC value of 0.75.

### Analysis of Dataset B3

In this study, dataset B3 included a total of 444 bitewing radiographs obtained from 237 patients, including 138 females and 99 males, with a mean age of 58.41 ± 11.04 years. The ROI in each radiographic image was selected, resulting in 260 images of maxillary teeth and 184 images of mandibular teeth. These images covered a range of dental types, with 7 anterior teeth, 90 premolar teeth, and 347 molar teeth. The dataset B3 was divided into two groups based on the type of intrabony defect present: 205 images were classified as three-wall intrabony defects, while 239 images were categorized as non-three-wall intrabony defects.

**Fig. 4:**
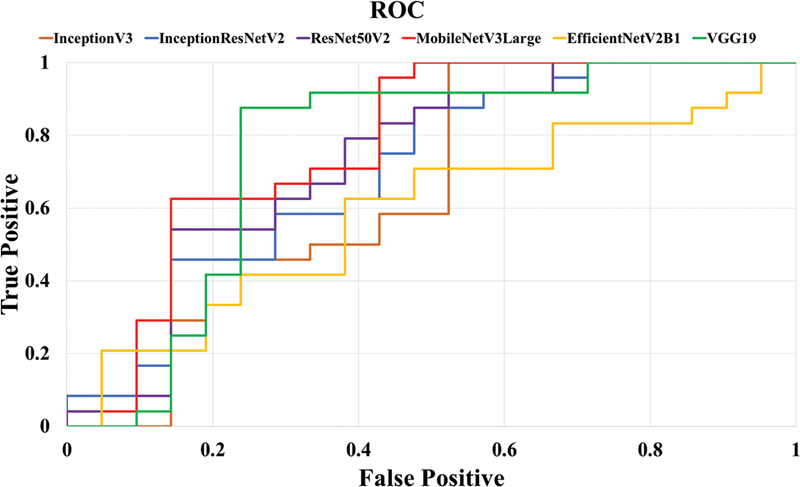
Representative curves of dataset B3 for training by six CNN models, ROC curves were characterized by a true positive rate on the *Y* -axis, and false positive rate on the *X* -axis. The areas under ROC curves or AUC from each dataset were calculated. AUC, area under curve; CNN, convolutional neural network; ROC, receiver operating characteristic.

According to Table 3 and Fig. 4 , the results of training dataset B3 with six different models are as follows: the AUC reflected the ability of the proposed model to distinguish between three-wall intrabony defects and non-three-wall intrabony defects. MobileNetV3Large achieved the highest AUC value of 0.77, indicating excellent discrimination, followed by VGG19 with an AUC value of 0.75.

**Table 3:** Model evaluation of dataset B3

Model	AUC	Accuracy	Precision	Recall	Specificity	NPV	F1
InceptionV3	0.66	0.71	0.83	0.48	0.92	0.67	0.61
InceptionResNetV2	0.70	0.67	0.69	0.52	0.79	0.66	0.59
ResNet50V2	0.73	0.69	0.77	0.48	0.88	0.66	0.59
MobileNetV3Large	0.77	0.69	0.71	0.57	0.79	0.68	0.63
EfficientNetV2B1	0.59	0.53	0.00	0.00	1.00	0.53	0.00
VGG19	0.75	0.78	0.82	0.67	0.88	0.75	0.74

Abbreviation: AUC, area under curve.

## Discussion

This study pioneered using a CNN model to classify three-wall intrabony defects in intraoral radiographs. Surgical procedures confirmed the accuracy of this classification. High model performances were indicated in InceptionResNetV2, ResNet50V2, MobileNetV3large, and VGG19 of dataset B3. Dataset B3, which excluded circumferential defects and was derived from a vertical bitewing radiograph, achieved a high AUC score of 0.75, a recall of 0.67, and a specificity equal to 0.88 for the VGG19 model. The high specificity of VGG19 suggested that it could classify non-three-wall defects better than three-wall intrabony defects. Lee et al utilized the VGG19 model to predict teeth with periodontal issues. A high AUC value was observed for premolars (0.828) and molars (0.734). 20 Previously, two-dimensional intraoral radiographs had an accuracy of 0.25 for assessing three-wall intrabony defects. 11 However, intraoral radiographs were ineffective in detecting three-wall intrabony defects, with a sensitivity of only 0.22, while CBCT scans had a sensitivity of 0.66. 30 According to our study, a CNN model might improve the classification and interpretation techniques for three-wall intrabony defects.

CNNs have shown remarkable accuracy in other dental applications, including identifying dental implant types from intraoral radiographs. This further supports our findings regarding the capability of CNN models to classify intrabony defects. 31 The performance of CNN models can be classified into multiple categories based on the specific conditions of each dataset. Accuracy evaluation factors have been adjusted for, including defects in circumferential, buccal, or lingual regions, tooth types, and intraoral techniques. However, it is important to note that intraoral radiographs have certain limitations when used on superimposed defects, such as those found in circumferential defects or defects located on the buccal or lingual surfaces. 32 It is possible to improve the performance of models by adjusting the factors that affect the data. After such adjustments, our models achieved an AUC value ≥ 0.7 in datasets B1, B3, C1, C3, and C4. Additionally, our study found that the vertical bitewing technique showed excellent performance when using the CNN model in datasets B3 and C3. However, an earlier study did not observe any significant impact of the periapical or bitewing technique on detecting dental restorations by the model's accuracy. 33

Dataset C4 achieved the highest performance in model training with an AUC value of 0.79. However, overfitting is possible as this dataset, consisting of 288 radiographic images, was designed to classify three-wall intrabony defects in the anterior and premolar teeth, which are less common than in the molar area. 34 This might limit its applicability to broader conditions in the anterior and premolar areas.

The limitation of this study was the input conditions for adjustment and model training, which resulted in smaller image quantities within each dataset, which could be improved by augmenting datasets with more images. Retrospective design constraints were imposed on the intra- and intercalibration of periodontists. Technological limitations impacted model performance. AI technologies, including CNN models, are already playing a key role in improving diagnostic accuracy across various oral diagnostic applications, including periodontal disease, which aligns with our findings. 35 To comprehensively classify three-wall intrabony defects, evaluating the CNN model for exceptional and combined three-wall defects is needed. Integrating radiographic images with clinical data can improve periodontal diagnosis and treatment planning.

## Conclusion

Our research focused on classifying three-wall intrabony defects in periodontal radiographs using various CNN models. We refined our datasets under specific conditions and optimized the models using vertical bitewing images. The best-performing models, MobileNetV3Large and VGG19, showed promising results, strong classification metrics, and significant implications for enhancing periodontal surgery diagnostics. Further integration and evaluation of these models in clinical practice could improve patient care and early detection of intrabony defects.
